# Supplementary material for: Molecular basis for plasma membrane recruitment of PI4KA by EFR3
Source: Sci Adv. 2024 Dec 20;10(51):eadp6660. doi: 10.1126/sciadv.adp6660 (PMC11661448; doi:10.1126/sciadv.adp6660)
Supplement: Supplementary file 1 — Figs. S1 to S6 Tables S1 and S2 Legend for data S1 [file sciadv.adp6660_sm.pdf]

Science Advances
AAAS

Supplementary Materials for

Molecular basis for plasma membrane recruitment of PI4KA by EFR3

Sushant Suresh *et al.*

Corresponding author: Calvin K. Yip, calvin.yip@ubc.ca; John E. Burke, jeburke@uvic.ca

Sci. Adv. **10**, eadp6660 (2024)
DOI: 10.1126/sciadv.adp6660

The PDF file includes:

Figs. S1 to S6
Tables S1 and S2
Legend for data S1

Other Supplementary Material for this manuscript includes the following:

Data S1

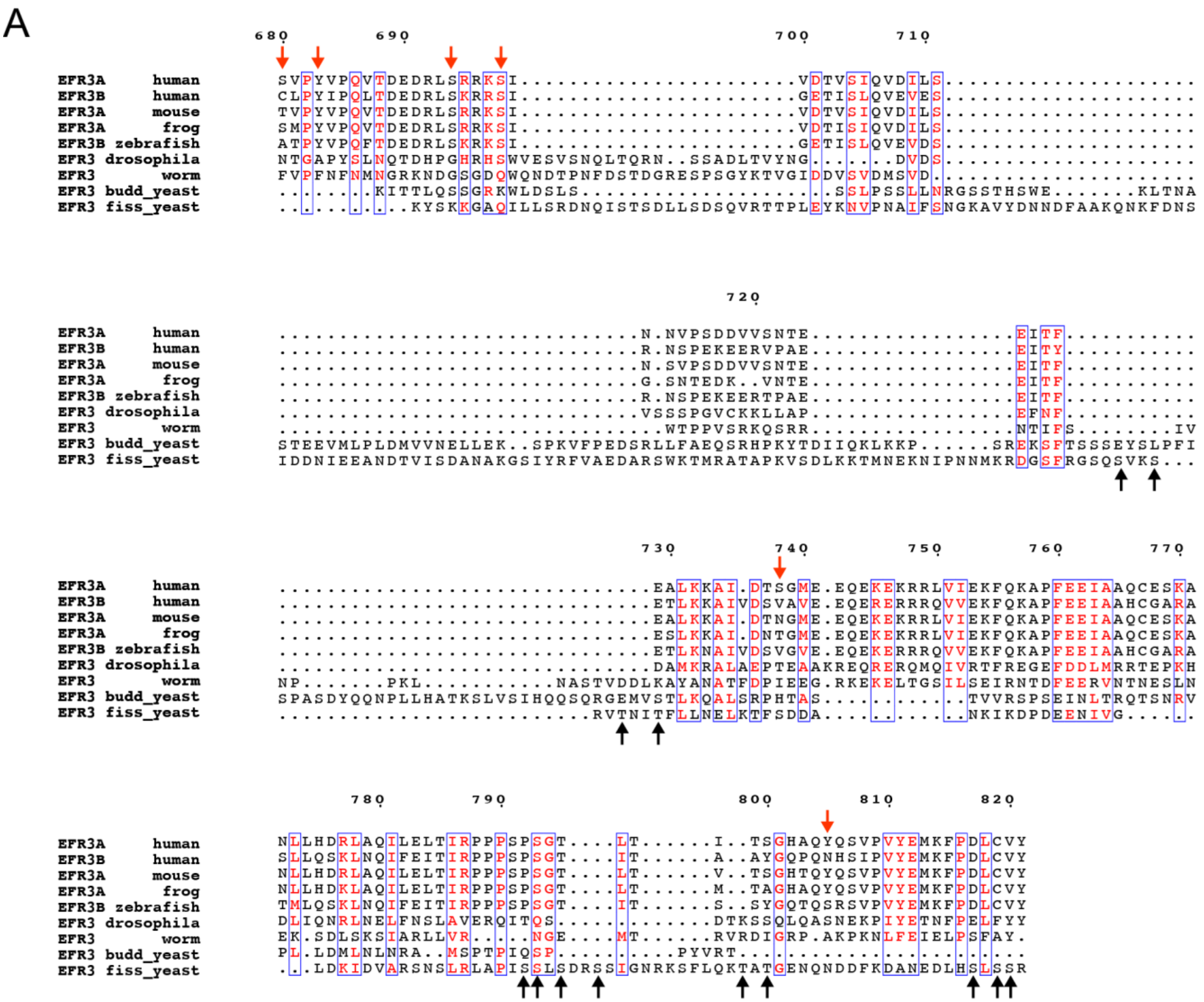

Figure S1: EFR3 C-terminal tail sequence alignment

A. Multiple sequence alignment (generated with ESPript 3.0) of EFR3 from *Homo sapiens, Mus musculus, Xenopus laevis, Danio rerio, Drosophila melanogaster, Caenorhabditis elegans*, *Schizosaccharomyces pombe,* and *Saccharomyces cerevisiae.* Human EFR3A residues that are reported phosphorylation sites (PhosphoSite) are annotated above in red. Yeast Efr3 residues that are reported phosphorylation sites (PhosphoGRID) are annotated below in black.

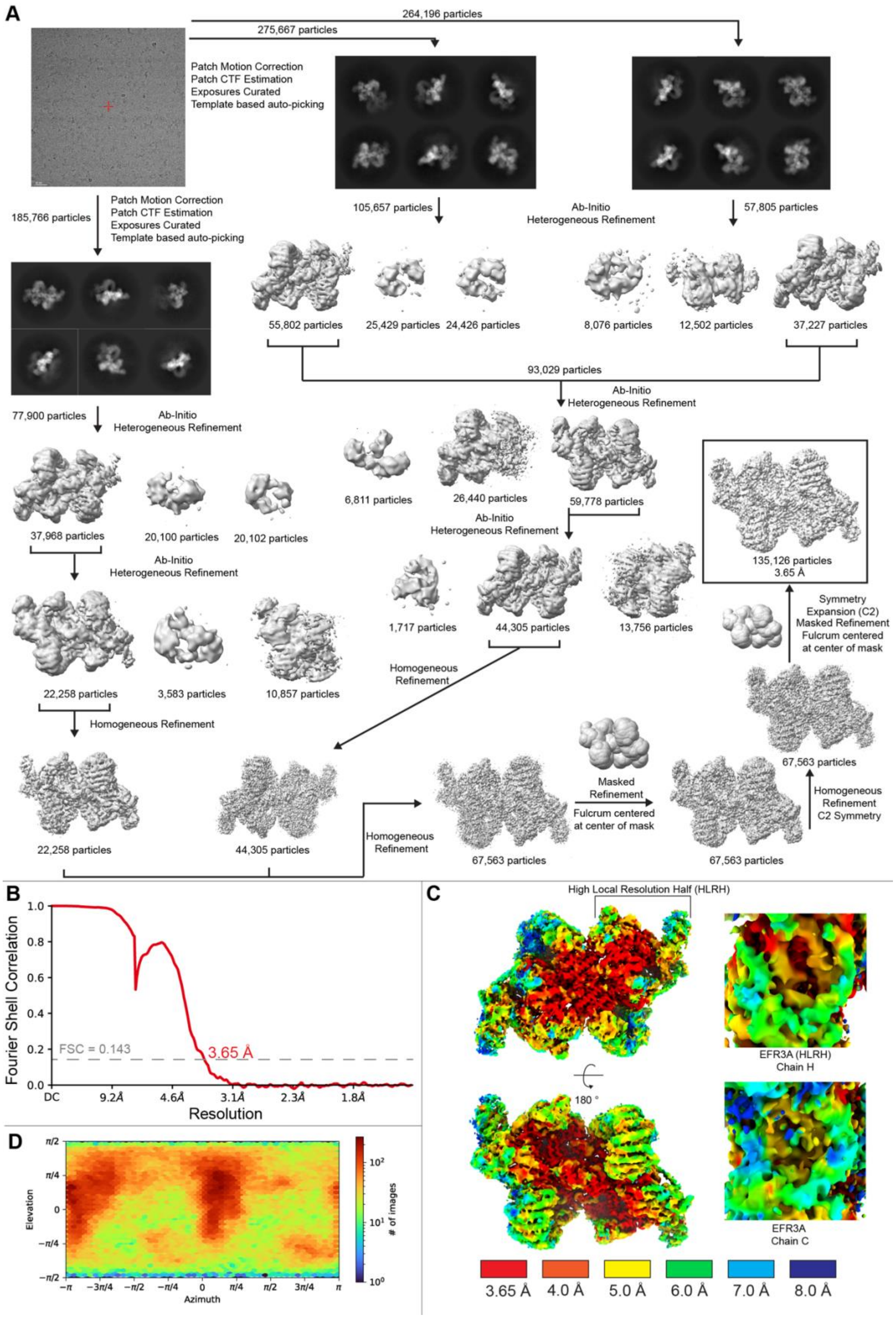
A
264,196 particles
275,667 particles
Patch Motion Correction
Patch CTF Estimation
Exposures Curated
Template based auto-picking
185,766 particles
Patch Motion Correction
Patch CTF Estimation
Exposures Curated
Template based auto-picking
105,657 particles
Ab-Initio
Heterogeneous Refinement
57,805 particles
55,802 particles
25,429 particles
24,426 particles
8,076 particles
12,502 particles
37,227 particles
93,029 particles
Ab-Initio
Heterogeneous Refinement
77,900 particles
Ab-Initio
Heterogeneous Refinement
6,811 particles
26,440 particles
59,778 particles
37,968 particles
20,100 particles
20,102 particles
Ab-Initio
Heterogeneous Refinement
Ab-Initio
Heterogeneous Refinement
135,126 particles
3.65 Å
1,717 particles
44,305 particles
13,756 particles
Symmetry
Expansion (C2)
Masked Refinement
Fulcrum centered
at center of mask
22,258 particles
3,583 particles
10,857 particles
Homogeneous
Refinement
Homogeneous Refinement
67,563 particles
Masked
Refinement
Fulcrum centered
at center of mask
Homogeneous
Refinement
C2 Symmetry
22,258 particles
44,305 particles
Homogeneous
Refinement
67,563 particles
67,563 particles
B
Fourier Shell Correlation
FSC = 0.143
3.65 Å
DC
9.2Å
4.6Å
3.1Å
2.3Å
1.8Å
Resolution
C
High Local Resolution Half (HLRH)
180 °
EFR3A (HLRH)
Chain H
EFR3A
Chain C
3.65 Å
4.0 Å
5.0 Å
6.0 Å
7.0 Å
8.0 Å
D
Elevation
Azimuth
# of images

Figure S2 (related to Fig.1): Cryo-EM data processing
A. Cryo-EM data processing workflow showing a representative micrograph from screening on the 200 kV Glacios, representative 2D class averages, and the image processing strategy used to generate a 3D reconstruction of the PI4KA/TTC7B/FAM126A/EFR3A complex.
B. Gold standard Fourier shell correlation coefficient (FSC) curve after auto tightening by cryoSPARC for the final map.
C. Final map coloured according to local resolution estimated using cryoSPARC v4.2.1, with a zoom-in of the EFR3A density.
D. Viewing direction distribution plot of particles in the final cryo-EM reconstruction output by cryoSPARC v4.2.1.

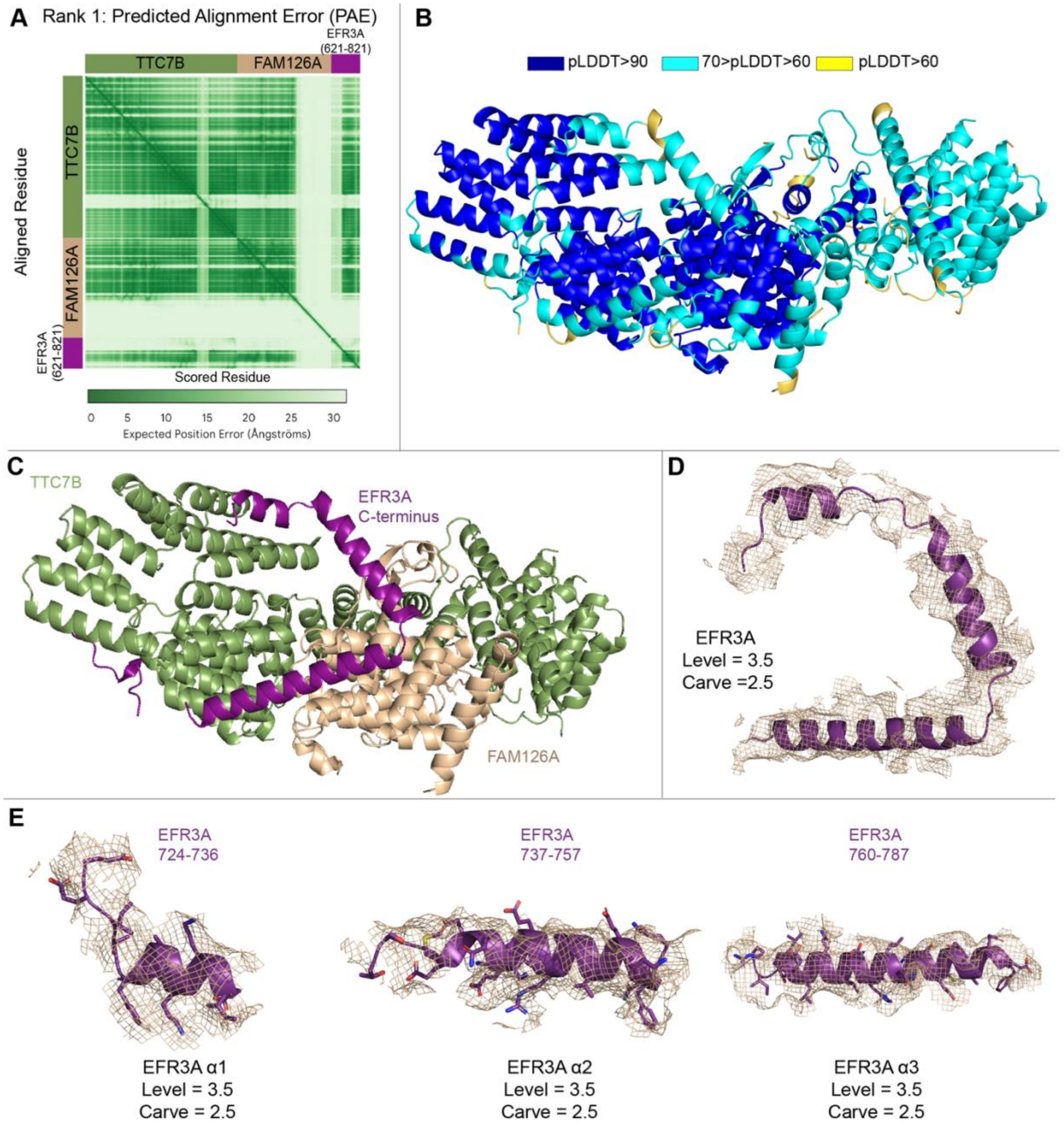

Figure S3: Model building with AlphaFold3 and map to model density fit

A. Predicted aligned error (PAE) of the AlphaFold3 prediction of the TTC7B-FAM126A and EFR3A C-terminus.

B. AlphaFold3 model of TTC7B-FAM126A in complex with the EFR3A C-terminus with the per-residue confidence metric predicted local-distance difference test (pLDDT) <60 removed, coloured according to pLDDT score.

C. AlphaFold3 model of TTC7B-FAM126A in complex with EFR3A C-terminus with the per-residue confidence metric predicted local-distance difference test (pLDDT) <60 removed.

D. Electron density of EFR3A C-terminus (Chain H).

E. Electron density of selected regions of EFR3A (Chain H). (L) EFR3A α1 with density for EFR3A (724-736) shown, (M) EFR3A α2 with density for EFR3A (737-759), and (R) EFR3A α3 with density for EFR3A (760-787) shown.

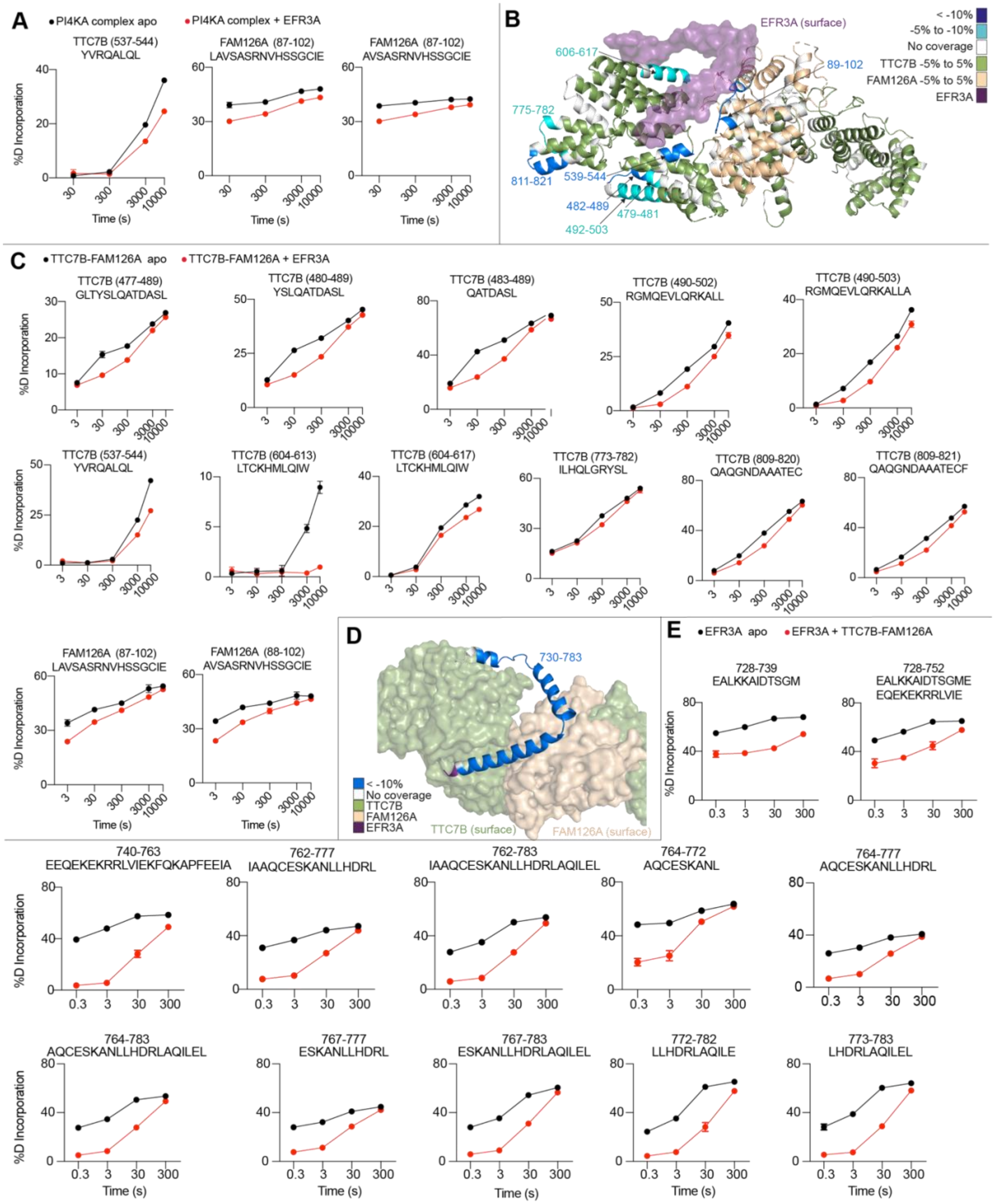

Figure S4: Raw HDX-MS deuterium incorporation curves

A. Relative deuterium incorporation traces for peptides showing significant differences in deuterium exchange (defined as >5%, >0.45 Da, and p<0.01 in an unpaired two-tailed t-test at any time point) upon PI4KA complex binding to EFR3A.

B. Significant differences in deuterium exchange (defined as >5% >0.45 Da, and p<0.01 in an unpaired two-tailed t-test at any time point) upon TTC7B-FAM126A dimer binding to EFR3A mapped on the high resolution half of our structural model with all regions annotated.

C. Relative deuterium incorporation traces for peptides showing significant differences in deuterium exchange (defined as >5%, >0.45 Da, and $p<0.01$ in an unpaired two-tailed t-test at any time point) upon TTC7B-FAM126A dimer binding to EFR3A.
D. Significant differences in deuterium exchange (defined as >5%, >0.45 Da, and $p<0.01$ in an unpaired two-tailed t-test at any time point) upon MBP-EFR3A binding to the TTC7B-FAM126A dimer mapped on the high resolution half of our structural model with all regions annotated.
E. Relative deuterium incorporation traces for peptides showing significant differences in deuterium exchange (defined as >5%, >0.45 Da, and $p<0.01$ in an unpaired two-tailed t-test at any time point) upon MBP-EFR3A binding to the TTC7B-FAM126A dimer.

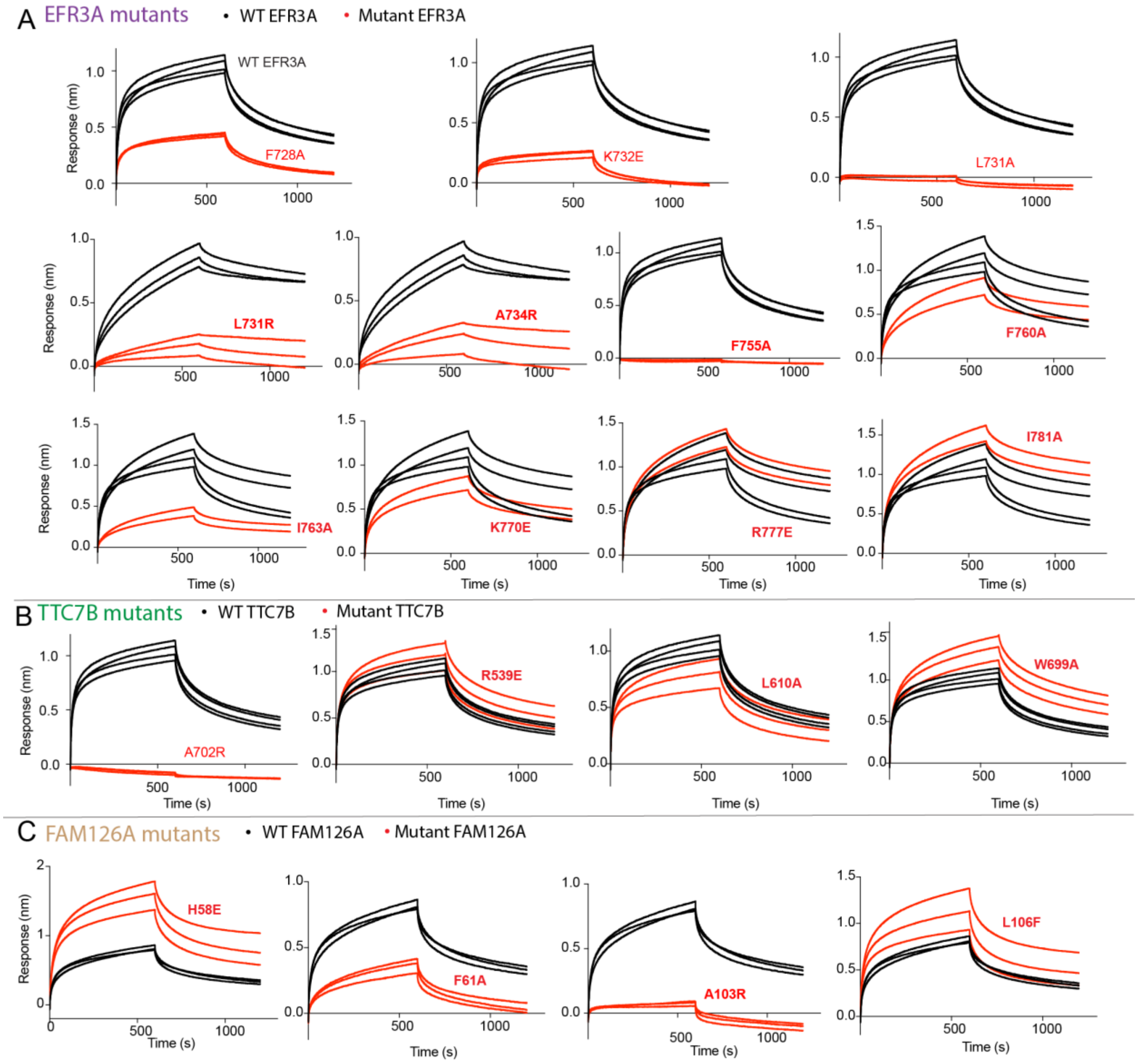

Figure S5: BLI raw data

A. Raw BLI association and dissociation curves of all EFR3A mutants (shown in table S2.) compared to WT. His-EFR3A was loaded on the anti-penta-His tip at 200 nM and dipped in TTC7B-FAM126A at 500 nM.

B. Raw BLI association and dissociation curves of all TTC7B mutants (shown in table S2.) compared to WT. His-EFR3A was loaded on the anti-penta-His tip at 200 nM and dipped in TTC7B-FAM126A at 500 nM.

C. Raw BLI association and dissociation curves of all TTC7B mutants (shown in table S2.) compared to WT. His-EFR3A was loaded on the anti-penta-His tip at 200 nM and dipped in TTC7B-FAM126A at 500 nM.

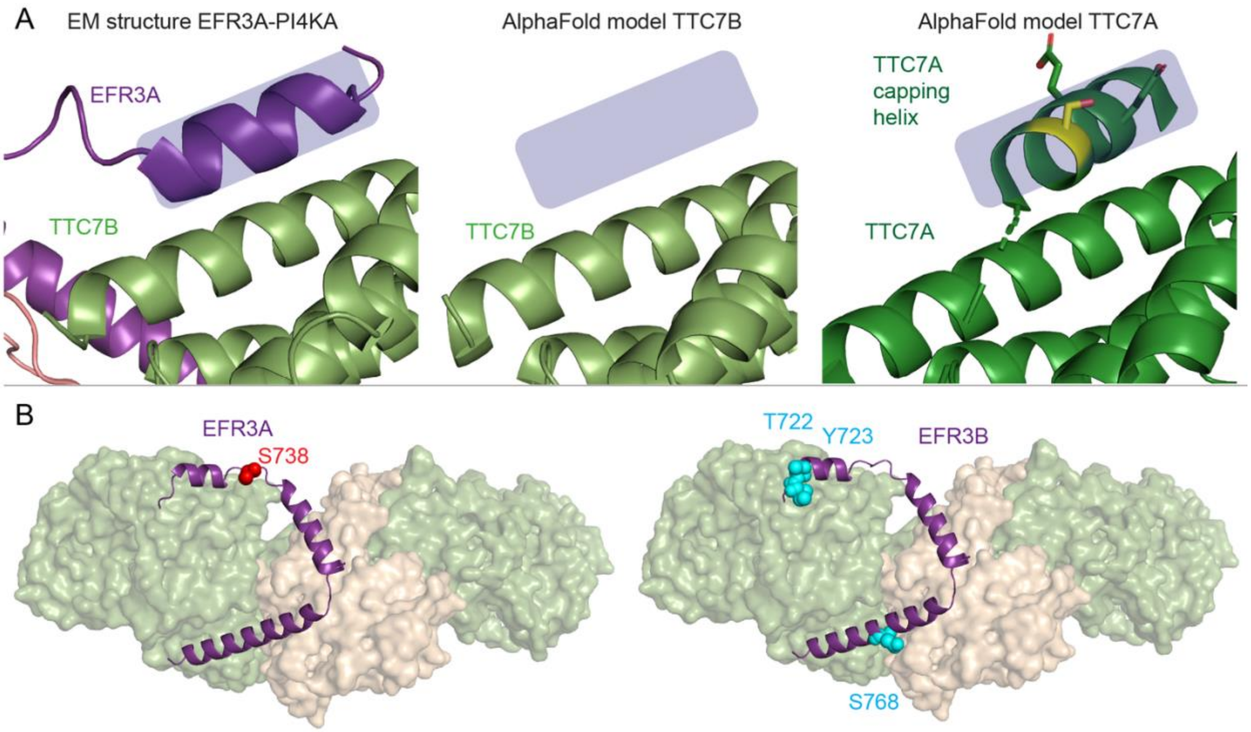

Figure S6: Differences between TTC7A/TTC7B and post-translational modification sites present at the EFR3-TTC7-FAM126 interface

A. The structure of the α1 helix of EFR3A bound to TTC7B is shown on the top left. The AlphaFold models of TTC7B (middle) and TTC7A (right) are shown with all very low confidence regions (pLDDT values less than 50 being removed). AlphaFold predicts a capping helix in TTC7A at this binding interface, which has a reported phosphorylation site (PhosphoSite) at S690 (colored yellow in the right panel). Additional reported TTC7A phosphorylation sites (PhosphoSite) near this site are in predicted unstructured regions including S647, S678, S679, and T693.

B. (L) EFR3A and (R) EFR3B phosphorylation sites (PhosphoSite) mapped on the cryo-EM model of PI4KA complex-EFR3A C-terminus.

Table S1 Cryo-EM data collection, refinement, and validation statistics

	PI4KA/TTC7B/FAM126A (1-308)-EFR3A C-terminus EMD- 44413 PDB:9BAX
**Data collection and processing**	
Magnification	165,000
Voltage (kV)	300
Electron exposure (e/ Å^2)	50
Defocus range (µM)	0.5-2.0
Pixel size (Å)	0.77
Symmetry imposed	C2
Initial particle images (no.)	725,629
Final particle images (no.)	67,563
Map resolution (Å)	3.65
FSC threshold	0.143
Map resolution range (Å)	3.3-10
**Refinement**	
Initial model used (PDB)	6BQ1/AlphaFold3
Model Resolution (Å)	3.65
FSC threshold	0.143
Map sharpening B factor	-22.55
Model composition	
Non-hydrogen atoms	42100
Protein residues	5264
Ligands	0
B-factors	
Protein	208.37
Validation	
Mol probability score	1.68
Clashscore	6.36
Poor rotamers (%)	1.17
Ramachandran	
Favoured	96.02
Allowed	3.95
Outliers	0.04
R.M.S. deviations	
Bond lengths (Å)	0.002
Bond angles (°)	0.500

Table S2 Key Reagent/Resources

REAGENT or RESOURCE	SOURCE	IDENTIFIER

**Bacterial and virus strains**		
E.coli XL10-GOLD KanR Ultracompetent Cells	Agilent	200317
E.coli DH10EMBacY Competent Cells	Geneva Biotech	DH10EMBacY
C41(DE3) chemically competent cells	Lab stock	
**Chemicals, peptides, and recombinant proteins**		
Deuterium Oxide 99.9%	Sigma Aldrich	151882-10X1ML
BS^3	Thermo Fischer Scientific	21580
ATP	Sigma	A7699-1g
$MgCl_2$	Caledon Laboratory Chemicals	4720-1
**Deposited Data**		
Mass spectrometry proteomics data	https://www.ebi.ac.uk/pride/	PXD043442
PDB	https://www.rcsb.org/	9BAX
EMDB	https://www.emdataresource.org/	44413
**Recombinant DNA**		
PI4KA Complex	(*21*)	GD177
EFR3A (721-791)	This paper	SS77
MBP-EFR3A (721-791)	This paper	MJ319
EFR3A (F728A)	This paper	SS88
EFR3A (L731A)	This paper	SS99
EFR3A (L731R)	This paper	SS140
EFR3A (L734R)	This paper	SS141
EFR3A (K732E)	This paper	SS89
EFR3A (F755A)	This paper	SS90
EFR3A (F760A)	This paper	SS91
EFR3A (I763A)	This paper	SS102
EFR3A (K770E)	This paper	SS92
EFR3A (R777E)	This paper	SS93
EFR3A (I781A)	This paper	SS94
TTC7B-FAM126A (1-308) WT	(*21*)	GD176
TTC7B-FAM126A (1-308) WT – *E.coli*	This paper	AS29
TTC7B (A702R)-FAM126A (1-308)	This paper	MS8
TTC7B (R539E)-FAM126A (1-308)	This paper	SS115
TTC7B (L610A)-FAM126A (1-308)	This paper	SS113
TTC7B (W699A)-FAM126A (1-308)	This paper	SS116
TTC7B-FAM126A (H58E) (2-308)	This paper	SS104
TTC7B-FAM126A (F61A) (1-308)	This paper	SS103
TTC7B-FAM126A (A103R) (1-308)	This paper	MS4
TTC7B-FAM126A (L106F) (1-308)	This paper	SS105
EFR3Bha_gsgT2A_TTC7Bmyc_FlagFam126A	This paper	C3
EFR3Bha_t2a_TTC7B myc_p2a_Flag FAM126A (A103R)	This paper	NH74
EFR3Bha_t2a_TTC7B (A702R) myc_p2a_Flag FAM126A	This paper	NH75

EFR3Bha Δc-term (1-715)_gsgT2A_TTC7Bmyc_FlagFam126A	This paper	NH93
EFR3Bha(L726A, F751A)_gsgT2A_TTC7Bmyc_FlagFam126A	This paper	NH94
EGFP-PI4KA	(*42*)	
L10-mVenus-tPT2A-nLuc-PI4KA	This paper	PM-PI4KA^{BRET}
**Software and algorithms**		
cryoSPARC v4.2.1	Structura Bio	https://cryosparc.com/
Phenix-1.19.1	Open source	https://phenix-online.org/
COOT-0.9.4.1	CCP4	https://www2.mrc-lmb.cam.ac.uk/personal/pemsley/coot/
HDExaminer	Sierra Analytics	http://massspec.com/hdexaminer
Bruker Compass DataAnalysis 4.2	Bruker	http://www.bruker.com
GraphPad Prism 7	GraphPad	https://www.graphpad.com
PEAKS 7	Bioinformatics Solutions Inc.	https://www.bioinfor.com/versions/
FragPipe (v19.1)	Nesvizhskii Lab – University of Michigan	fragpipe.nesvilab.org
Adobe Illustrator 2021	Adobe	https://www.adobe.com/products/illustrator.html
ESPript 3.0	SBGrid consortium	https:espript.ibcp.fr
PyMOL	Schroedinger	http://pymol.org
ChimeraX	UCSF	https://www.cgl.ucsf.edu/chimerax/
Modelangelo	Scheres Lab - MRC	https://github.com/3dem/model-angelo
**Other**		
C-flat Holey Thick Carbon Grid 2.0 µm hole 1.0 µm space 300 mesh	Electron Microscopy Studies	CFT312-100
SF9 insect cells for expression	Expression Systems	94-001S
Octet HIS1K Biosensors	Sartorius	18-5120
Enzymate Protein Pepsin Column, 300Å, 5µm, 2.1 mm X 30 mm	Waters	186007233
ProDx Pepsin Column F 10-32	Trajan Scientific Americas	359997870
ACQUITY UPLC BEH C18 1.7 µm, 2.1 mm x 5 mm	Waters	186004629
ACQUITY UPLC Peptide BEH C18 Column, 300Å, 1.7 µm, 100 mm X 2.1 mm	Waters	186003686

Other Supplementary Material for this manuscript includes the following:

Data S1: Source data.

This document includes all raw, processed and statistical analysis of HDX-MS data corresponding to Fig 2 and fig S4; Raw SEC traces corresponding to Fig 1 and Fig 2; Raw and processed BLI data corresponding to Fig 3, Fig 4 and fig S5; Model-map FSC corresponding to Fig 1, fig S2 and fig S3; SDS-PAGE gels representing proteins used in this manuscript.
